# Associations between prisons and recidivism: A nationwide longitudinal study

**DOI:** 10.1371/journal.pone.0267941

**Published:** 2022-05-17

**Authors:** Rongqin Yu, Niklas Långström, Mats Forsman, Arvid Sjölander, Seena Fazel, Yasmina Molero

**Affiliations:** 1 Department of Psychiatry, University of Oxford, Oxford, United Kingdom; 2 Department of Medical Epidemiology and Biostatistics, Karolinska Institutet, Stockholm, Sweden; 3 National Board of Health and Welfare, Stockholm, Sweden; 4 Department of Clinical Neuroscience, Karolinska Institutet, Stockholm, Sweden; University of Bern, SWITZERLAND

## Abstract

**Objectives:**

To examine differences in recidivism rates between different prisons using two designs—between-individual and within-individual—to account for confounding factors.

**Methods:**

We examined recidivism rates among 37,891 individuals released from 44 Swedish prisons in three security levels, and who were followed from 2006 to 2013. We used longitudinal data from nationwide registers, including all convictions from district courts. First, we applied a between-individual design (Cox proportional hazards regression), comparing reconviction rates between individuals released from prisons within the same security level, while adjusting for a range of individual-level covariates. Second, we applied a within-individual design (stratified Cox proportional hazards regression), comparing rates of reconviction within the same individuals, i.e., we compared rates after release from one prison to the rates in the same individual after release from another prison, thus adjusting for all time-invariant confounders within each individual (e.g. genetics and early environment). We also adjusted for a range of time-varying individual-level covariates.

**Results:**

Results showed differences in the hazard of recidivism between different prisons in between-individual analyses, with hazards ranging from 1.22 (1.05–1.43) to 4.99 (2.44–10.21). Results from within-individual analyses, which further adjusted for all time-invariant confounders, showed minimal differences between prisons, with hazards ranging from 0.95 (0.87–1.05) to 1.05 (0.95–1.16). Only small differences were found when violent and non-violent crimes were analyzed separately.

**Conclusions:**

The study highlights the importance of research designs that more fully adjust for individual-level confounding factors to avoid over-interpretation of the variability in comparisons across prisons.

## Introduction

Each year, around 30 million people enter and leave prison worldwide [1]. Among former prisoners at least one in five is reconvicted, and in many countries fifty percent of those released from prisons are reconvicted within 2 years [2, 3]. Research examining the risk of recidivism has traditionally focused on individual-level risk factors, such as mental disorders [4–6], substance misuse [7, 8], and previous criminal history [9]. The role of system-level factors, such as prisons, has been less researched. It has been suggested that certain prisons may increase the risk of reoffending beyond individual-level risk factors [10, 11] because they differ in important factors that could affect recidivism, such as availability of rehabilitation programs or mental health services [12, 13]. Comparisons between different prisons–e.g. high-security vs. open prisons, or public vs. private prisons–remain inconclusive, with studies showing both different [12–18] and similar [10, 19–21] risks of repeat offending.

However, previous research includes a major methodological limitation; although studies have controlled for several individual-level factors associated with re-offending (e.g. gender, age, crime history, and psychiatric disorders [22–24], they have not accounted for all selection effects. That is, individuals sentenced to different prisons may vary on unmeasured individual-level risk factors associated with recidivism [25]. This may confound results, and previously reported associations could therefore be spurious, pointing to a need for research designs that more fully adjust for unmeasured individual-level risk factors [26]. Prior studies have used a between-individual design when comparing prisons, i.e. they have compared rates of recidivism between different individuals. We propose using a within-individual study design where each individual is compared to him/herself, thus acting as their own control across time. This design accounts for important individual-level risk factors that are constant within each individual, such as genetics and early environment, and adjusts further for unmeasured individual-level factors that may confound associations.

Using a design that examines differences in recidivism with better precision could provide important implications for the management of offenders, criminal justice policy, and service development to reduce recidivism; research findings pointing to differences in recidivism rates between prisons could inform prevention and intervention efforts targeting prison-level factors, and improve resource allocation within the prison system. On the other hand, findings that indicate no differences between prisons would suggest that resources should be allocated to manage individual-level risk factors for recidivism, rather than to change prison-level factors.

### Aims

This study set out to test whether there were differences in recidivism between different prison facilities using two sets of designs: First, we used a standard design that compared reoffending rates between different individuals (‘between-individual design’) while adjusting for a range of measured sociodemographic, criminological, and psychiatric individual-level factors. Subsequently, we used a design that compared rates of repeat offending for the same person following being placed in different prisons (‘within-individual design’). By comparing each individual to themselves, the within-individual design inherently adjusts for all time-invariant individual-level confounding factors (i.e. factors that do not change over the follow-up period). In addition, we also controlled for a range of measured sociodemographic, criminological, and psychiatric covariates individual-level time-varying individual-level confounding factors (i.e. factors that may change over the follow-up period).

These analyses were adopted to test whether recidivism rates were different by which prison someone was placed in or allocated to. Namely, whether different prisons—in different locations, supervised by different staff members, and with other varying characteristics—were associated with different reoffending risks when applying between and within-individual models, respectively. However, the analyses did not attempt to identify which specific factors within these prisons that were associated with reoffending risks, if differences were to be found. We used validated, high-quality register data and applied both designs in a large nationwide cohort that included all individuals released from all prisons in Sweden during a period of eight years.

## Methods

### Study design and participants

We examined reoffending rates among 37,891 individuals placed in prison in Sweden during the period from 1 January 2006 to 31 December 2013. We identified individuals using data from nationwide Swedish registers, including the Total Population Register [27], the National Crime Register [28], the Prisoner Register [29], the Patient Register [30], and the Cause of Death Register [31]. These registers were linked using the unique ten-digit personal identification number assigned to all residents in Sweden. All data were pseudonymized, and ethical approval was received from the Regional Research Ethics Committee at Karolinska Institutet (2013/5:8), which waived the right for informed consent.

### Measures

#### Prisons

The study included all prisons in Sweden operating during the study period, and information was retrieved from the Prison and Probation Register. In Sweden, prisons are divided into three security levels; level 1 have the highest level of security, followed by level 2 (medium) and 3 (low) [32]. Before the start of a prison sentence, a risk and needs assessment is carried out by the Prison and Probation Service [33]. In this assessment, several different aspects are considered; risk of recidivism and escape, type and severity of index crime, criminal ties to or conflicts with other prisoners, as well as vocational, educational and treatment needs like substance misuse. Assessment results determine the choice of prison security level and specific prison. Assessments are based on documentation from prior and current convictions and pre-trial evaluations with personal contact with the offender; addressing personal history, living circumstances and security intelligence available to the Prison and Probation Service. Naturally, conditions vary between prisons, also slightly across units within the same security level. In general, overall possibilities to monitor and control prisoners determines the prison security level. Access to education, work or treatment needs is not dependent on security level but varies somewhat with local circumstances. Overall, one national Prison and Probation Service and strongly rehabilitation-oriented legislation, regulations and practice make such opportunities reasonably well provided across all Swedish prisons (https://www.kriminalvarden.se/swedish-prison-and-probation-service/rehabilitation/). Almost all prisoners are released on parole after having served 2/3 of their sentences. Professionals at local probation department offices of the National Prison and Probation Service throughout Sweden then supervise the parolee, usually for 12 months, and coordinate continued rehabilitation efforts. These efforts could involve support from social services, outpatient addiction treatment services and recidivism-reducing programs, psychiatric care, and vocational training or education.

#### Recidivism

Recidivism was defined as reconviction of any crime after release from prison. Information on crimes was obtained from the National Crime Register, which includes all convictions in Swedish district courts. This register has total national coverage—only 0.05% of all registered convictions had incomplete personal identification numbers [34]. Conviction data included persons who received custodial (including forensic psychiatric care) or noncustodial sentences. Conviction data also included fines, and cases in which the prosecutor issued a summary sanction order (usually including a fine) or a waiver of prosecution. Summary sanction orders and waivers of prosecution can be used in minor cases after a full investigation, and by accepting them, the offender admits guilt and full prosecution is not needed. However, they are registered as convictions in the National Crime Register in the same manner as if the offender had been convicted in court. Furthermore, we carried out sensitivity analyses stratified on violent crimes (i.e. crimes against people, including attempted, completed, and aggravated forms of murder, manslaughter, unlawful threats, harassment, robbery, arson, assault, assault on an official, kidnapping, stalking, coercion, and all sexual offences except purchase of sexual services), and non-violent crimes (i.e. all other offences) as in previous work [35]. In additional sensitivity analyses, we examined only custodial sentences.

#### Covariates

We adjusted for measured confounding factors that are directly or indirectly related to both prison type and criminal recidivism [36], including sex, age at the start of prison sentence, number of previous convictions, imprisonment length, alcohol and drug use disorders, as well as psychiatric disorders [22, 23, 37]. We collected information on sex and age at the start of placement or reception in each prison from the Total Population Register. We retrieved information on the number of previous convictions (both prison, probation, and community service), imprisonment length, and current alcohol and drug use disorders from the Prison and Probation Register. We collected information on all psychiatric diagnoses (International Classification of Diseases 10^th^ revision [ICD-10] codes F00-F99) [38] from the National Patient Register, which contains data on all diagnoses assigned in inpatient and specialist outpatient care [30]. Swedish register-based psychiatric diagnoses generally have moderate to high concordance rates with clinical diagnoses [30] with high rates for severe mental illnesses, such as schizophrenia and bipolar disorder [39]. In depression, a recent study found good reliability [4].

### Statistical analyses

Follow-up started on the day an individual was released from prison (earliest 1 January 2006), and ended on the date of reoffending, migration from Sweden, death, or at the end of follow-up (31 December 2013), whichever occurred first. If a person was re-imprisoned during the study period, a new follow-up began on the day they were released from that prison.

To examine the link between prisons and recidivism, we performed two different analyses. First, we applied a *between-individual design* using Cox proportional hazards regression. Here, we estimated the hazard of reoffending after prison release between individuals; that is, we compared the rate of reoffending in individuals released for example from prison A to the rate of reoffending in individuals released from prison B. We adjusted for correlated data (i.e. that the same individual could contribute with several observations) by computing the robust sandwich covariance matrix estimate [40]. We also adjusted for measured covariates that may confound associations between prison type and reoffending, including sex, age at the start of new placement or reception in a prison, number of previous convictions, imprisonment length, alcohol and drug use disorders, and psychiatric disorders. Mathematically, the model is given by

$$\lambda (t_{ij}|P_{ij},X_{ij})=\lambda_{0}(t_{ij})e^{\beta P_{ij}+\gamma X_{ij}}$$

where *t_ij_*, *P_ij_* and *X_ij_* are the time until reoffending, the prison, and the vector of measured covariates for the *j*: th prison visit within the *i*:th individual, respectively, $\lambda (t_{ij}|P_{ij},X_{ij})$ is the conditional hazard function at time *t_ij_*, given *P_ij_* and *X_ij_*, and *λ*_0_(*t_ij_*) is an unspecified baseline hazard.

Second, we employed a *within-individual design* using stratified Cox proportional hazards regression. This was done because estimates in the between-individual design may be susceptible to unmeasured confounders that affect both the selection into prison category (and sometimes individual prison within category) and reoffending. In the within-individual design, each individual is entered as a separate stratum in the analysis, thus acting as their own control [41]. We compared the rate of reoffending in an individual when he/she was released for example from prison A to the rate of reoffending in the same individual when he/she was released from prison B. The rate ratio of reoffending was thus implicitly adjusted for all time-invariant confounders within each individual—whether these confounders were measured or unmeasured—such as genetics, early family environment, and potentially changeable factors that remained stable during the follow-up (e.g., continuous alcohol or drug abuse throughout the follow-up) [42, 43]. Furthermore, we adjusted for measured time-varying confounding factors, that is, factors that may change during follow-up, including age, number of previous convictions, imprisonment length, alcohol and drug use disorders, and psychiatric disorders. In the between-individual Cox proportional hazards regression, all individuals in the sample contribute to the estimate of recidivism. In the within-individual stratified Cox proportional hazards regression, only individuals who have different exposures (i.e., are released from different prisons within the same security level) and experience at least one event (i.e., they reoffend) contribute directly to the estimate. All other individuals contribute indirectly through the association of time-varying covariates with the outcome.

During follow-up, 35.9% (n = 2,098) of the individuals in high security prisons had different exposures (among those, the mean number of exposures was 2.1, standard deviation [SD] = 1.3), 32.2% in medium security prisons (n = 7,775) had different exposures (mean = 2.9, SD = 1.3), and 11.7% (n = 2,187) in low security prisons had different exposures (mean = 2.2, SD = 0.6). See S1 Table for background characteristics on individuals who had one vs. multiple exposures.

Mathematically, the model is given by

$$\lambda (t_{ij}|P_{ij},X_{ij},individual\;i)=\lambda_{0i}(t_{ij})e^{\beta^{*}P_{ij}+\gamma^{*}X_{ij}}$$

where $\lambda (t_{ij}|P_{ij},X_{ij},individual\;i)$ is the conditional hazard function at time *t_ij_*, given *P_ij_*, *X_ij_* and the individual *i*. By conditioning on the individual, the model implicitly adjusts for all time-invariant confounders within the individual; these are absorbed by the individual-specific baseline hazard on the right-hand side of the model formulation. The Cox proportional hazards regression estimates incidence ratios, thus automatically adjusting for differences in the length of follow-up [44]. We tested the proportional hazards assumption for both the between- and within-individual design by using a Schoenfeld residual-based test, and detected no substantial violations of this assumption.

In all analyses, prisons were stratified by the three different security levels; high security (‘level 1’; 7 prisons), medium security (‘level 2’; 27 prisons), and low security (‘level 3’; 10 prisons). This was done to control for unmeasured differences in time-varying individual-level risk factors associated with allocation to a specific security level [18]. Only offences committed after prison release from each specific security level contributed to the rate of offending within that level. For example, if an individual was released from a medium security prison and reoffended, this offence contributed to the rate of recidivism in medium security prisons. If the individual was re-imprisoned in a high security prison, any reoffence after release from this prison contributed to the rate of recidivism in high security prisons. During the study period, 74.8% (n = 28,347) were placed in only one of the security levels; 22.0% (n = 8,323) were allocated to two different security levels, and; 3.2% (n = 1,221) had placements in all three security levels. Five prisons included both security levels 2 and 3 (i.e., units that belonged to level 2, as well as units belonging to level 3). Since it was not possible to determine which specific unit the individual had been placed in, and the majority of units within these five prisons belonged to the medium security level (i.e., level 2), we classified these prisons as medium security prisons. In sensitivity analyses, we excluded the five prisons from the medium security level. We also carried out sensitivity analyses where we classified the five prisons as belonging to the low security level (i.e., level 3).

In the analyses, all prisons were compared individually to a reference prison within the same security level. To select the reference prison, a Cox proportional hazards regression was first performed with a randomly selected prison as the reference prison. The prison with the lowest hazard ratio in this analysis was then selected as the reference prison in all subsequent analyses (this was done separately for all three security levels).

Around a quarter of sentences were spent in two or more different prisons (24.8%, 13,821/55,655 sentences). Each such sentence was therefore “cloned”, so that the sentence contributed with one placement (or record) per prison to the analysis. Because placements in prisons that constitute a larger proportion of the sentence may have had a greater impact on reoffending than placements that constitute a smaller proportion, each placement or allocation in prison was weighted based on how long the individual had been placed in that specific prison. The linear weighting was based on the percentage of the total sentence the individual had been placed or allocated in a specific prison. For example, if an individual had a 100-day sentence, of which 75 days had been in prison A, and 25 days in prison B, the placement for prisons A and B were given weights equal to 0.75 and 0.25, respectively, in the analysis. We also carried out sensitivity analyses where we excluded ‘cloned’ sentences, i.e. where the individual served their sentence in two or more different prisons within the same security level.

## Results

In total, we included 37,891 individuals (91.9% males) who had served 55,655 prison sentences during an average follow-up period of 8 years (Table 1). Of those, 5,850 individuals served their sentence in a high security prison, 24,150 served in a medium security prison, and 18,656 served in a low security prison during follow-up. Mean number of prison sentences per person during the study period was 1.7 (standard deviation [SD] = 1.3). The average age at the start of the first prison sentence was 37.5 years (SD = 12.9), and the average length of imprisonment was 138.6 days (SD = 188.4). During the study period, 53.3% (n = 20,202) of individuals reoffended at least once after prison release; 50.0% (n = 18,943) of the cohort committed a non-violent crime, and 16.2% (n = 6,153) committed a violent crime. Furthermore, 22.4% (n = 8,492) were convicted to a custodial sentence, 10.0% (n = 3,751) were convicted to a non-custodial sentence, and 33.2% (n = 12,555) were convicted to another type of sentence. For more cohort characteristics, and characteristics stratified by prison security level, see Table 1.

**Table 1:** Characteristics of a cohort of 37,891 prisoners followed 2006 to 2013 in Sweden.

Sex	All prisons (n = 37,891)	High security prisons (level 1) (n = 5,850)^†^	Medium security prisons (level 2) (n = 24,150)^†^	Low security prisons (level 3) (n = 18,656)^†^
Male	91.9% (34,810)	98.7% (5,775)	88.9% (21,475)	96.5% (18,000)
Female	8.1% (3,081)	1.3% (75)	11.1% (2,675)	3.5% (656)
Prison placement				
Mean age at the start of the first placement in prison during the study period (SD)	37.5 (12.9)	33.2 (10.8)	35.8 (11.6)	40.7 (13.5)
Mean number of days in prison per sentence (SD)	138.6 (188.4)	211.3 (287.9)	141.6 (176.4)	103.2 (143.8)
Mean number of prison sentences during the study period (SD)	1.7 (1.3)	1.7 (1.3)	1.9 (1.5)	1.3 (0.8)
Number of reconvicted individuals				
Any crime	53.2% (20,202)	54.1% (3,162)	61.7% (14,894)	44.7% (8,339)
Non-violent crime	50.0% (18,943)	51.7% (3,022)	59.6% (14,402)	41.8% (7,790)
Violent crime	16.2% (6,153)	22.2% (1,298)	23.5% (5,665)	13.1% (2,449)
Type of reconvictions^††^				
Custodial sentences	22.4% (8,492)	13.7% (803)	24.7% (5,976)	13.1% (2,435)
Non-custodial sentences	10.0% (3,751)	3.6% (209)	10.8% (2,597)	5.4% (1,006)
Other sentences	33.2% (12,555)	14.6% (849)	32.6% (7,852)	22.9% (4,273)
Number of reconvicted criminal events^†††^				
Any crime	43,959	5,289	28,197	10,473
Non-violent crime	41,943	5,035	27,139	9,769
Violent crime	13,889	2,136	8,942	2,811
Mental health problems				
Alcohol use disorders	33.9% (12,849)	34.7% (2,032)	36.2% (8,731)	32.3% (6,023)
Drug use disorders	44.1% (16,714)	58.7% (3,436)	59.2% (14,352)	25.7% (4,801)
Psychiatric disorders	11.5% (4,350)	14.1% (822)	14.3% (3,448)	7.6% (1,420)

Notes

^**†**^ Individuals could be placed in two or more security levels during the same prison sentence or during different sentences, consequently, the combined number of individuals in the three different security levels is larger than the total number of individuals in the study

^**††**^ Other sentences include summary sanction orders, waivers of prosecution, and fines. Individuals may be reconvicted to more than type of sentence during follow-up

^**†††**^ Non-violent and violent crimes that were committed on the same day count as one event. Consequently, the number of any crime is lower than the number of violent and non-violent crimes combined.

### Hazard of any recidivism among individuals placed in high security (level 1) prisons

**Fig 1:**
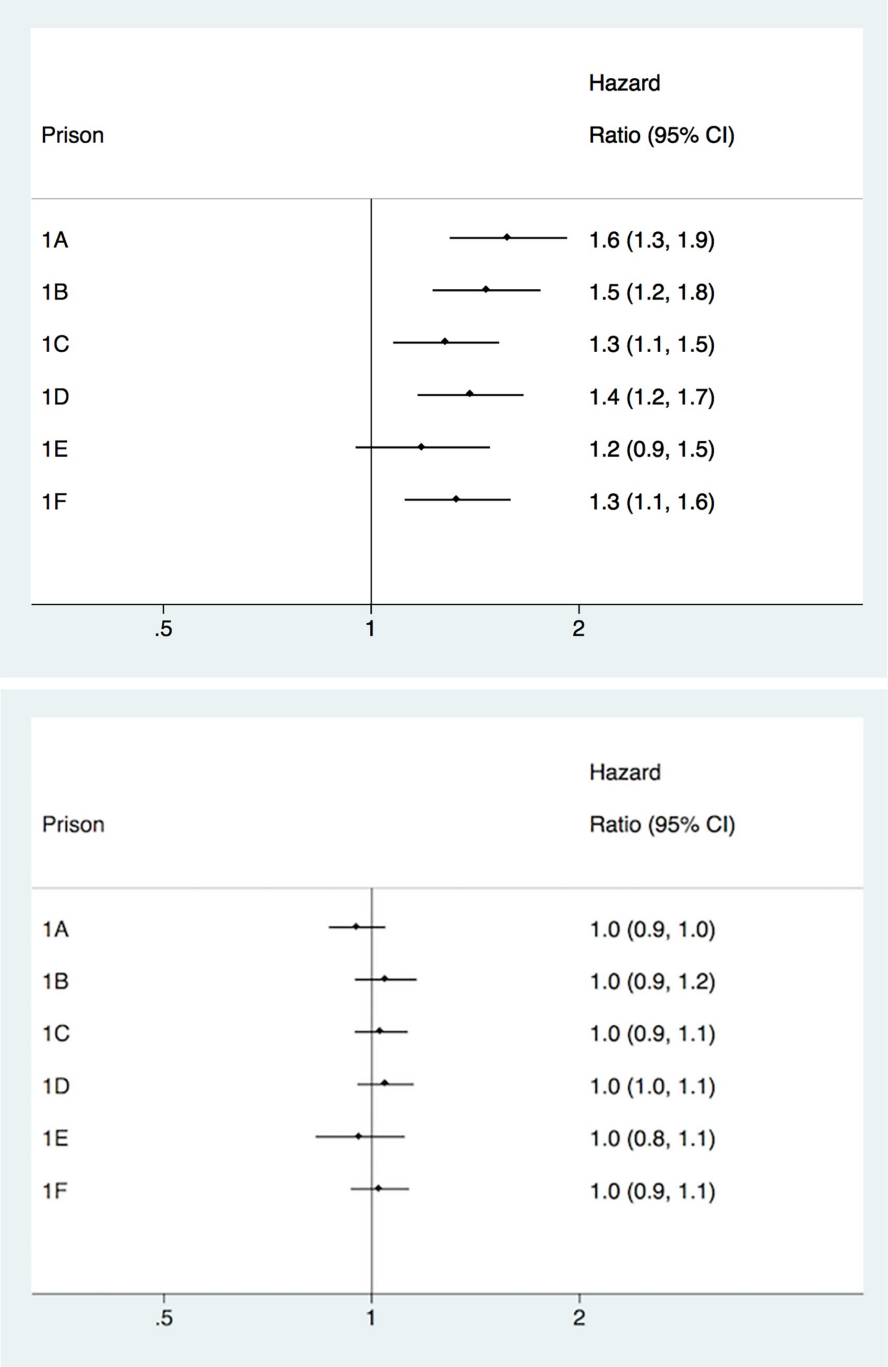
**A.** Between-individual analyses of recidivism risk among prisoners released from high security prisons (level 1). **B.** Within-individual analyses of recidivism risk among prisoners released from high security prisons (level 1).

In between-individual analyses, there were differences in the hazard of any recidivism between high security prisons (Fig 1A). When the reference prison was compared with the other prisons, all except one were associated with an increased hazard of recidivism, with hazard ratios [HRs] ranging from 1.28 (CI = 1.08–1.53) to 1.58, CI = 1.30–1.92 (for regression estimates of the individual-level covariates that were adjusted for in the model, see S2 Table).

Subsequently, we conducted within-individual analyses using stratified Cox regression (Fig 1B). These analyses suggested that the overall hazard ratio of recidivism decreased to negligible levels for all prisons, compared to the effect sizes obtained in between-individual analyses, with HRs ranging from 0.95 (0.87–1.05) to 1.05 (0.95–1.16).

### Hazard of any recidivism among individuals placed in medium security (level 2) prisons

**Fig 2:**
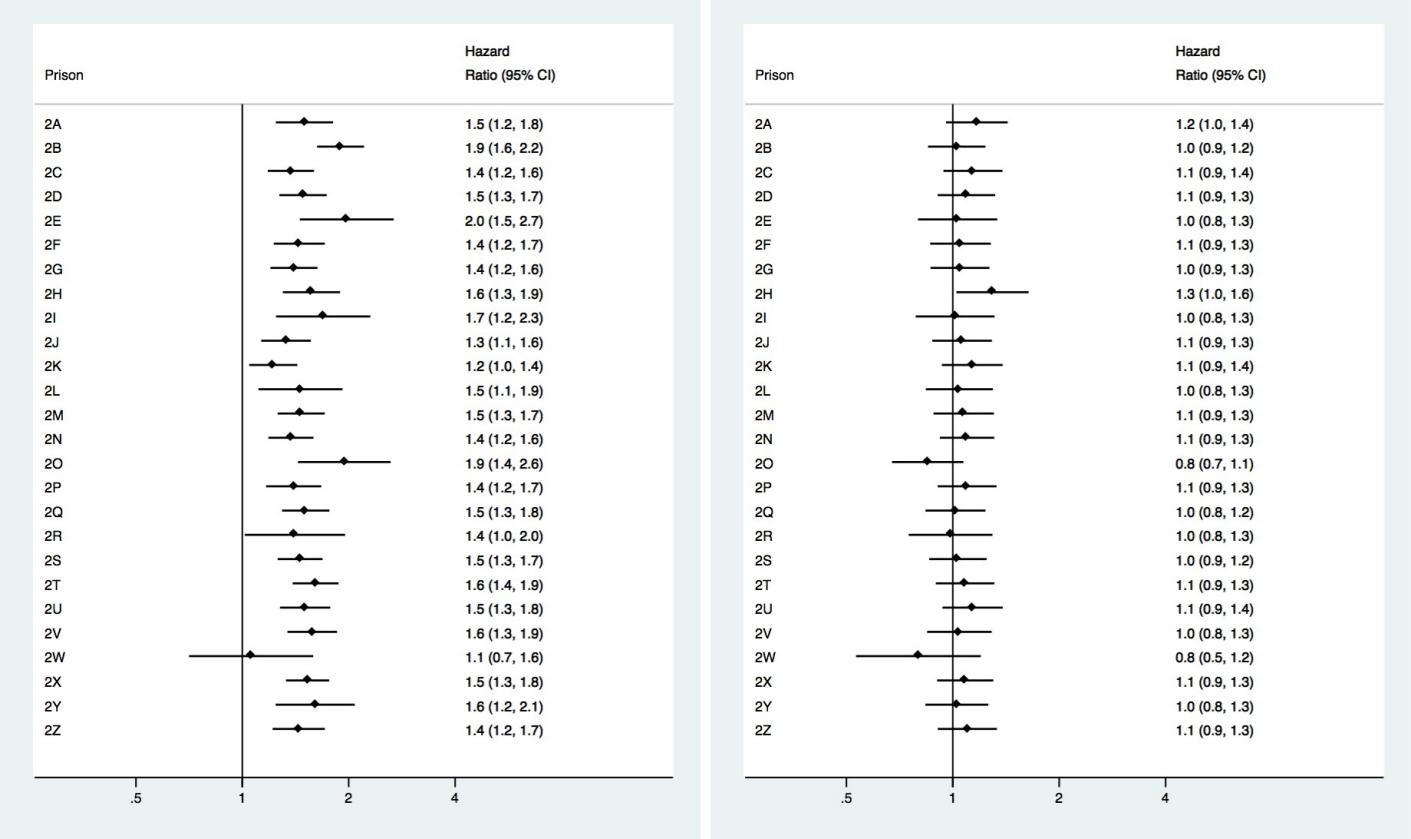
**A.** Between-individual analyses of recidivism risk among prisoners released from medium security prisons (level 2). **B.** Within-individual analyses of recidivism risk among prisoners released from medium security prisons (level 2).

For medium security prisons (Fig 2A), all prisons except one showed increased hazard ratio of any recidivism in between-individual analyses, with HRs ranging from 1.22 (1.05–1.43) to 1.98 (1.45–2.68). Again, in within-individual analyses (Fig 2B), differences in the hazard of recidivism disappeared, except for one prison (HR = 1.30, 1.02–1.63).

### Hazard of any recidivism among individuals placed in low security (level 3) prisons

**Fig 3:**
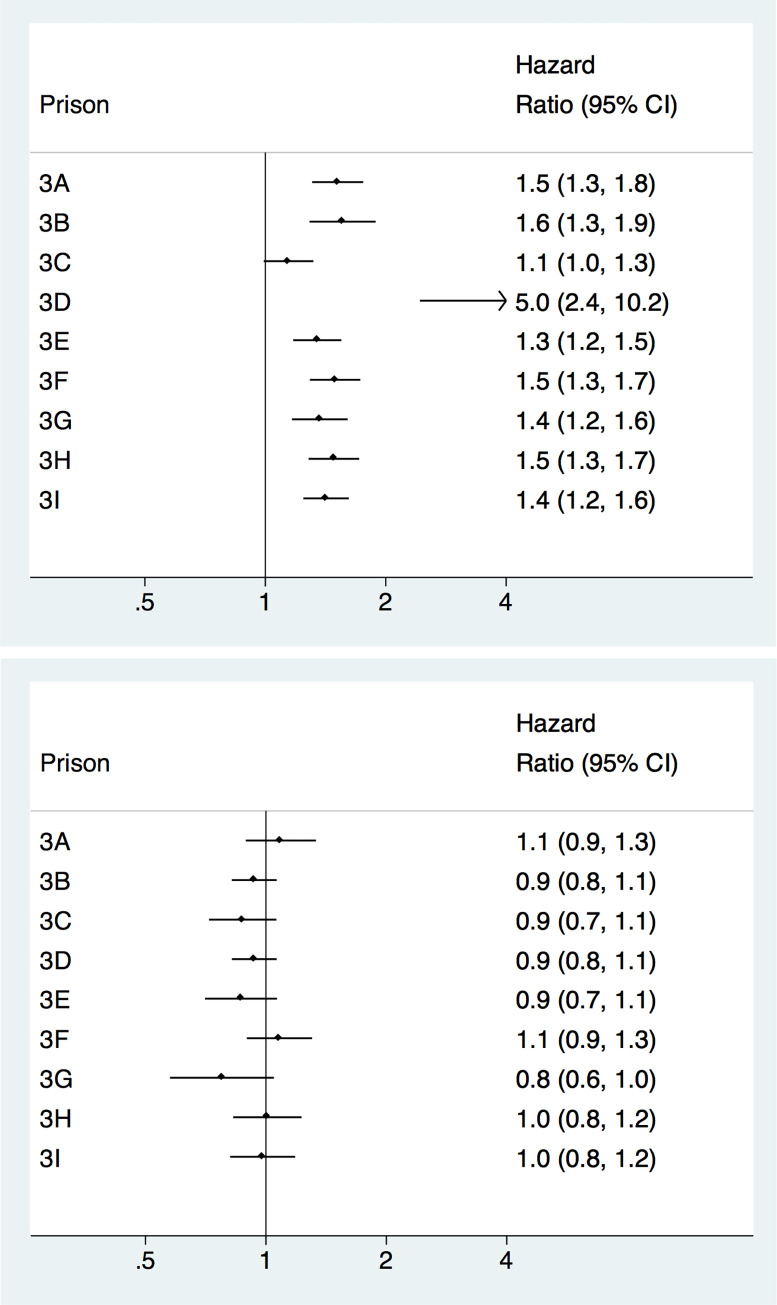
**A.** Between-individual analyses of recidivism risk among prisoners released from low security prisons (level 3). **B.** Within-individual analyses of recidivism risk among prisoners released from low security prisons (level 3).

In between-individual analyses of low security prisons (Fig 3A), all prisons except one showed increased hazards, with HRs ranging from 1.35 (1.17–1.55) to 4.99 (2.44–10.21). However, in within-individual analyses (Fig 3B), there were no meaningful differences between prisons.

### Sensitivity analyses

We carried out sensitivity analyses and examined associations separately for violent and non-violent recidivism. First, we addressed associations between prisons and *non-violent reoffending* for all three security levels. For high and low security prisons, results followed the overall patterns seen in the main analyses for any crime; that is, we found increased hazards of non-violent reconvictions for most prisons in between-individual analyses (S1A and S3A Figs), and no differences when comparing prisons in within-individual analyses (S1B and S3B Figs). In analyses of medium security prisons, we also found increased hazards of non-violent crimes in between-individual analyses for most prisons (S2A Fig). However, in contrast, we found decreased hazards for two prisons in within-individual analyses (S2B Fig).

Second, we carried out sensitivity analyses using *violent reoffending* as outcome. For high security prisons, we found increased hazards of violent reconvictions for two prisons in between-individual analyses (S4A Fig). These hazards were attenuated but remained increased in within-individual analyses. A third prison demonstrated an increased hazard in within-individual analyses (S4B Fig). For medium security prisons, we found increased hazards of violent recidivism for several prisons in between-individual analyses (S5A Fig), but no differences in within-individual analyses (S5B Fig). For low security prisons, we found increased hazards of violent crimes for two prisons in between-individual analyses (S6A Fig). Finally, in within-individual analyses, hazards were no longer increased for these two prisons (S6B Fig); however, we found a decreased hazard for another prison.

Third, we examined within-individual associations using *only custodial sentences* as outcome. Results suggested no differences between high security prisons (S7A Fig), increased hazards for eight medium security prisons (S7B Fig), and increased hazards for three low security prisons (S7C Fig).

Five prisons included both medium and low security level units. In our main analyses these were classified as medium security prisons. In sensitivity analyses, we excluded the five prisons from the analyses of the medium security prisons (S8 Fig). Results from the within-individual analyses remained similar to the main analyses of medium security prisons, i.e. showing no differences in the hazard of recidivism, except for one prison (HR = 0.73, 0.60–0.89). We also carried out sensitivity analyses where we classified the five prisons as low security prisons, examining them with all other low security prisons. Results from the within-individual analyses showed decreased hazards for three prisons (two of the with both low and medium security units), and no significant associations for the other prisons (S9 Fig).

Finally, we carried out within-individual analyses excluding those who had been placed in two or more different prisons within the same security level during the same sentence (i.e. ‘cloned’ sentences). These analyses included 5,131 individuals (of 5,850) in high risk prisons; 20,307 individuals (of 24,150) in medium risk prisons, and; 16,301 individuals (of 18,656) in low risk prisons. Results followed the same patterns as in the main analyses; showing no within-individual associations for high (S10A Fig), medium (S10B Fig), or low security prisons (S10C Fig).

## Discussion

We examined associations between offender placement in individual prisons and reoffending for 37,891 individuals with 20,202 reconvictions during a study period of 8 years. We compared all 44 prisons (7 high, 27 medium, and 10 low security prisons) in the entire country. First, we compared recidivism rates between individuals released from prisons within the same security level, while adjusting for a range of sociodemographic, criminological, and psychiatric covariates. Second, we compared rates of recidivism within the same individuals, that is, we compared rates after release from one prison to the rates in the same individual after release from another prison within the same security level. Recidivism rates were thus adjusted for all time-invariant confounders within each individual (e.g., genetics, perinatal conditions, early family environment, and factors that were stable during the follow-up). We also adjusted for a range of time-varying covariates that could change between prison placements or allocations within the same individual, including age, number of previous convictions, imprisonment length as well as substance use and psychiatric disorders. The results from the between-individual analyses suggested differences in the hazard of recidivism between prisons within all three security levels, with increased hazards ranging from 1.22 (1.05–1.43) to 4.99 (2.44–10.21). In within-individual analyses adjusted also for all time-invariant confounders within individuals, however, we found only minimal differences in recidivism rates between prisons with hazards ranging from 0.95 (0.87–1.05) to 1.05 (0.95–1.16). Still, we found a few differences in within-individual analyses when analyzing violent and non-violent reoffending, and reconvictions to custody separately.

### Interpretation of the results

Findings from the between-individual design pointed to differences in recidivism between prisons, while differences were attenuated or disappeared when individuals were compared to themselves following separate sentences within the same security category. Importantly, this suggests that between-individual analyses, while adjusted for several measured individual-level characteristics associated with reoffending, remained liable to residual confounding factors. By using a within-individual design, we controlled for all time-invariant confounding (measured or unmeasured), and our results indicate that observed variations in reconviction rates between prisons were largely due to differences between individuals, rather than differences between prisons. The differing results in the between and within-individual models could suggest that previous conclusions about differences in recidivism rates between prisons may, at least partly, be due to unobserved confounding [12, 15–17]. If this is the case, then the contribution of certain prison conditions to recidivism (e.g., climate) may have been overestimated. This would also suggest that it is important to target risk factors at the individual level, such as substance use disorders [45, 46], to reduce recidivism.

Our within-individual comparisons suggested that prisons within the same security level, in general, were similarly associated with overall recidivism, which could be attributed to consistency in programs, activities, and health services across prisons. During the last 15 years, most prisons run by the Swedish Prison and Probation Service have offered a range of evidence-based treatment programs to reduce criminal recidivism and substance misuse; programs that are reviewed and approved by an external scientific accreditation panel. In addition, all Swedish prisons have compulsory occupational activities, and offer the opportunity for studies [47]. Several manual-based programs, using cognitive behavioral therapeutic (CBT) theory and practice, are provided. These are similar to those employed in other high income countries, and are often transferred from, or inspired by, Canadian or UK approaches. The Swedish tax-funded general health services are available also to prisoners, to some extent complemented with nurses and consultant physicians, including psychiatrists, contracted specifically by the Prison and Probation Service. The consistency across prisons could have contributed to the small differences in recidivism rates between Swedish prisons. Further, prison sentences were shorter-term (mean: 139 days), which may limit the influence of prison placements or allocations on outcomes after release.

We found some differences between prisons when stratifying outcomes on violent and non-violent recidivism, and when examining reconvictions to custody. These differences may be associated with either time-varying selection mechanisms or with the specific features of these settings. Individuals released from two medium security prisons exhibited a decreased hazard of non-violent recidivism, although this could be a chance association due to multiple testing (the analyses compared 27 prisons). We also found some differences for violent recidivism among both high and low security prisons. This could suggest that differences between prisons may affect the rates of violent crime after prison release. Still, it was not possible to determine which specific factors that may have contributed to this association, as the effects of prison conditions and post-release environment were not examined in this study. On the other hand, our results could indicate that unmeasured time-varying confounding was still present, that is, that within-individual factors that changed between placements or allocations in different prison types confounded associations. Moreover, our analyses indicated that differences in recidivism rates, especially those from the between-individual analyses, applied mostly to non-violent reoffending, but not as much to violent reoffending, as there was more variability and larger confidence intervals in the analyses involving violent reoffending. This could be due to smaller sample sizes in the analyses of violent reoffending than for non-violent reoffending. It may also suggest that violent reoffending is less likely to be affected by prison conditions. More studies are needed to clarify whether differences between prisons may affect violent and non-violent offending differently.

We found some differences in recidivism rates between prisons in our study; for instance, while our analyses using custodial sentences as the outcome measure reported no differences for high security prisons, several of the medium and low security prisons were associated with increased hazards. This suggests that, although there were no large differences between prisons in overall reconvictions (including both custodial, non-custodial, and other sentences), there were differences in custodial reconvictions for medium and low security prisons. This may reflect a difference between prisons for more serious criminal recidivism. If these differences are replicated, then the contribution of specific prison conditions could be investigated. These may include availability to mental health services, primary care, psychotropic medications, the quality of interaction between inmate peers, positive relationships with prison officers, work opportunities, productive use of time, and access to well-conducted evidence-based training and treatment programs [12, 13, 48, 49].

### Strengths and limitations

This study has several advantages. We followed a total population of individuals released from all prisons in Sweden for eight years using validated, high-quality registers. To the best of our knowledge, this is the first study comparing differences in recidivism between individuals released from different prisons on a national level. In addition, the repeated measures of both exposures and outcomes allowed for within-individual comparisons, enabling us to control for important time-invariant confounders such as genetic and early environmental factors. Moreover, we also included time-varying factors which could change between placements in different prisons, such as substance use and psychiatric disorders, number of previous crimes, and crime severity (partly reflected by imprisonment length). The adjustments for a wide range of confounding factors allowed us to provide a more robust estimation of the link between prison and reoffending outcomes.

Caution is needed when interpreting the results. First, a within-individual analysis requires at least two observations per individual for comparisons. Therefore, only those released from prison at least twice during the study period contributed directly to the results (all other individuals contributed indirectly through the effect of covariates, such as age, on the estimate of the exposure, i.e., prison). This can result in selection bias, as individuals with at least two convictions might be different from those with only one. However, prevention work should prioritize individuals at high risk of recidivism [36], and those with multiple convictions usually constitute a high-risk group. Second, although our within-individual model inherently adjusted for all time-invariant factors, as well as observed time-varying factors entered separately as covariates, the model used by us did not adjust for unobserved time-varying factors. This could affect associations between prisons and recidivism. However, our results suggest that unobserved time-varying factors are unlikely to have had more than a marginal effect, as we only found small differences between prisons, mainly regarding serious recidivism. Third, national differences in prison and reconviction rates may affect statistical power and the generalizability of the present results: Sweden has lower prison population rates (59 per 100,000 of national population) as compared to the median rate for Western European countries (81/100,000) and the world (145/100,000) [50]. Sweden reports a 61% reconviction rate two years after prison release, while rates in other countries range between 20–63%, although direct comparisons are difficult due to substantial between-country variance in reconviction measures, and the use of non-custodial sentences for lower-risk offenders of less severe offences [3]. In addition, possibly due to the relatively consistent programming and services across Swedish facilities, within-person differences in recidivism rates were negligible across prison facilities. However, replications of this study in prison systems with more variation (such as in the U.S.) could yield different results. Therefore, replications and extensions of the findings in other countries are necessary. Fourth, the majority of within-individual analyses did not find differences in recidivism by prisons. This could mean that prisons had comparable conditions within the same security level, therefore, recidivism rates were similar between prisons. It could also mean that varying prison conditions, such as health services and ratio of prison guards to inmates, did not make a difference for recidivism rates. Alternatively, it could mean that varying prison conditions affected the risk of recidivism, but that the net result at facility-level was ambiguous due to differences in the strength and direction of these conditions. In the few instances where differences in recidivism rates were reported, such as violent reoffending risk on release from high security prisons, it was not possible to identify which factors within the prison system were linked to these differences. Therefore, the generalizability of the findings might be limited by the lack of examination of the effects of specific prison conditions. Finally, we did not examine post-release context, for instance, employment status [51], housing placement, post-release treatment and supervision [52], social ties [53], and neighborhood environment [54], which might be associated with recidivism.

## Conclusions

In a large national study of all 44 prisons in Sweden, we tested associations between prisons within each of three security levels and reoffending for 37,891 individuals. In between-individual analyses, adjusted for a range of measured sociodemographic, criminological and psychiatric factors, we found differences in recidivism between prisons. However, in the within-individual analyses, that adjusted for both time-invariant factors (such as genetics and early environment), and factors that could change between placements in different prisons (including age, substance use and psychiatric disorders, number of previous convictions, and imprisonment length as proxy of index crime severity), our findings indicated small differences in recidivism rates between prisons. This suggests that associations in the between-individual analyses were affected by unobserved confounding. Some small differences were, however, found in the within-individual analyses, which could be due to differences in prison conditions affecting serious reoffending, or to residual and unmeasured confounding by time-varying covariates.

By using both a standard design that compared reoffending rates between different individuals, and a design that compared rates of repeat offending within the same individual over time, this study highlights the importance of research designs that more fully adjust for individual-level risk factors when examining recidivism. This might be particularly important in order to avoid over-interpretation of outcome variability in comparisons across correctional or rehabilitation institutions.

## Supporting information

S1 Fig **A.** Between-individual analyses of recidivism risk among prisoners released from high security prisons (level 1): *Non-violent reoffending*. **B.** Within-individual analyses of recidivism risk among prisoners released from high security prisons (level 1): *Non-violent reoffending*.
(TIF)

S2 Fig **A.** Between-individual analyses of recidivism risk among prisoners released from medium security prisons (level 2): *Non-violent reoffending*. **B.** Within-individual analyses of recidivism risk among prisoners released from medium security prisons (level 2): *Non-violent reoffending*.
(TIF)

S3 Fig **A.** Between-individual analyses of recidivism risk among prisoners released from low security prisons (level 3): *Non-violent reoffending*. **B.** Within-individual analyses of recidivism risk among prisoners released from low security prisons (level 3): *Non-violent reoffending*.
(TIF)

S4 Fig **A.** Between-individual analyses of recidivism risk among prisoners released from high security prisons (level 1): *Violent reoffending*. **B.** Within-individual analyses of recidivism risk among prisoners released from high security prisons (level 1): *Violent reoffending*.
(TIF)

S5 Fig **A.** Between-individual analyses of recidivism risk among prisoners released from medium security prisons (level 2): *Violent reoffending*. **B.** Within-individual analyses of recidivism risk among prisoners released from medium security prisons (level 2): *Violent reoffending*.
(TIF)

S6 Fig **A.** Between-individual analyses of recidivism risk among prisoners released from low security prisons (level 3): *Violent reoffending*. **B.** Within-individual analyses of recidivism risk among prisoners released from low security prisons (level 3): *Violent reoffending*.
(TIF)

S7 Fig **A.** Within-individual analyses of recidivism risk among prisoners released from high security prisons (level 1); custodial sentences. **B.** Within-individual analyses of recidivism risk among prisoners released from medium security prisons (level 2); custodial sentences. **C.** Within-individual analyses of recidivism risk among prisoners released from low security prisons (level 3); custodial sentences.
(TIF)

S8 Fig Within-individual analyses of recidivism risk among prisoners released from medium security prisons (level 2), excluding five prisons with both medium and low security levels. (TIF)

S9 Fig Within-individual analyses of recidivism risk among prisoners released from low security prisons (level 3), including five prisons with both medium and low security levels. Note: * The prisons that have both security levels 2 and 3.
(TIF)

S10 Fig **A.** Within-individual analyses of recidivism risk among prisoners released from high security prisons (level 1); excluding cloned sentences. **B.** Within-individual analyses of recidivism risk among prisoners released from medium security prisons (level 2); excluding cloned sentences. **C.** Within-individual analyses of recidivism risk among prisoners released from low security prisons (level 3); excluding cloned sentences.
(TIF)

S1 Table Characteristics of individuals with one exposure (i.e. placement in one prison) vs. multiple exposures (i.e. placement in different prisons) within the same security level during 2006 to 2013 in Sweden. (DOCX)

S2 Table Covariate estimates in between- and within-individual analyses of recidivism risk among prisoners released from high, medium, and low security prisons (levels 1–3). * Reference category: Male; ** Only time-varying individual-level covariates (i.e. factors that may change during follow-up) are entered in the within-individual analyses.
(DOCX)
