# Supplementary figures and images for: Associations between prisons and recidivism: A nationwide longitudinal study
Source: PLoS One. 2022 May 17;17(5):e0267941. doi: 10.1371/journal.pone.0267941 (PMC9113604; doi:10.1371/journal.pone.0267941)

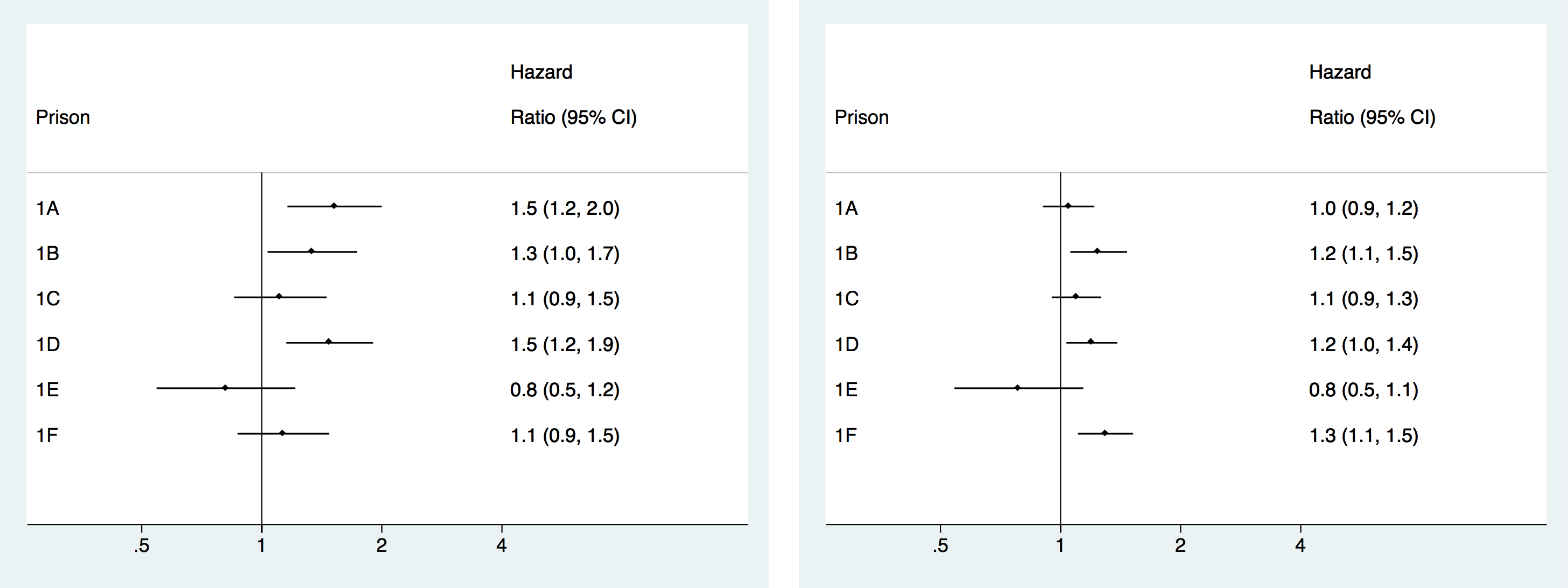

Supplement: S1 Fig — A. Between-individual analyses of recidivism risk among prisoners released from high security prisons (level 1): Non-violent reoffending. B. Within-individual analyses of recidivism risk among prisoners released from high security prisons (level 1): Non-violent reoffending. (TIF) [file pone.0267941.s001.tif]

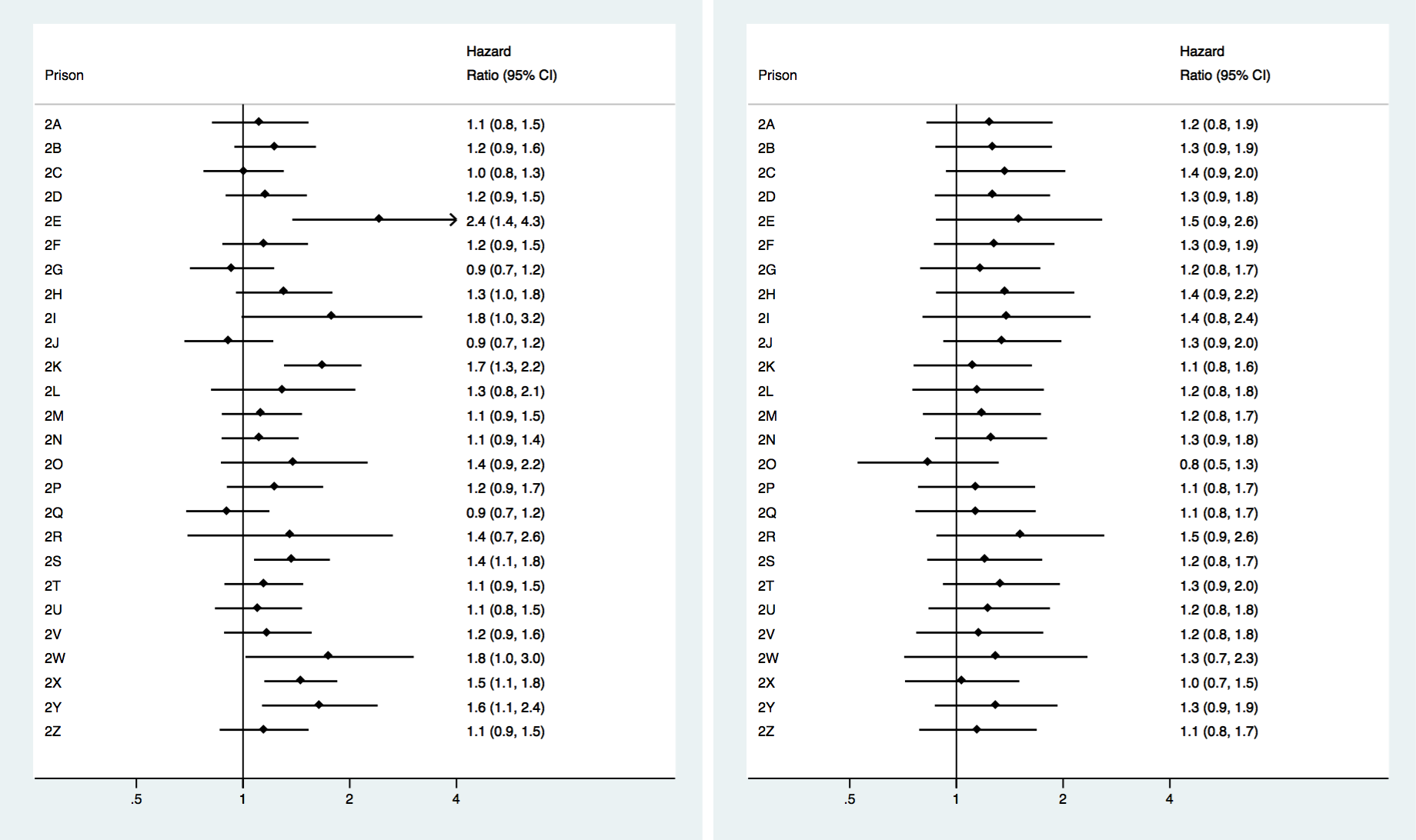

Supplement: S2 Fig — A. Between-individual analyses of recidivism risk among prisoners released from medium security prisons (level 2): Non-violent reoffending. B. Within-individual analyses of recidivism risk among prisoners released from medium security prisons (level 2): Non-violent reoffending. (TIF) [file pone.0267941.s002.tif]

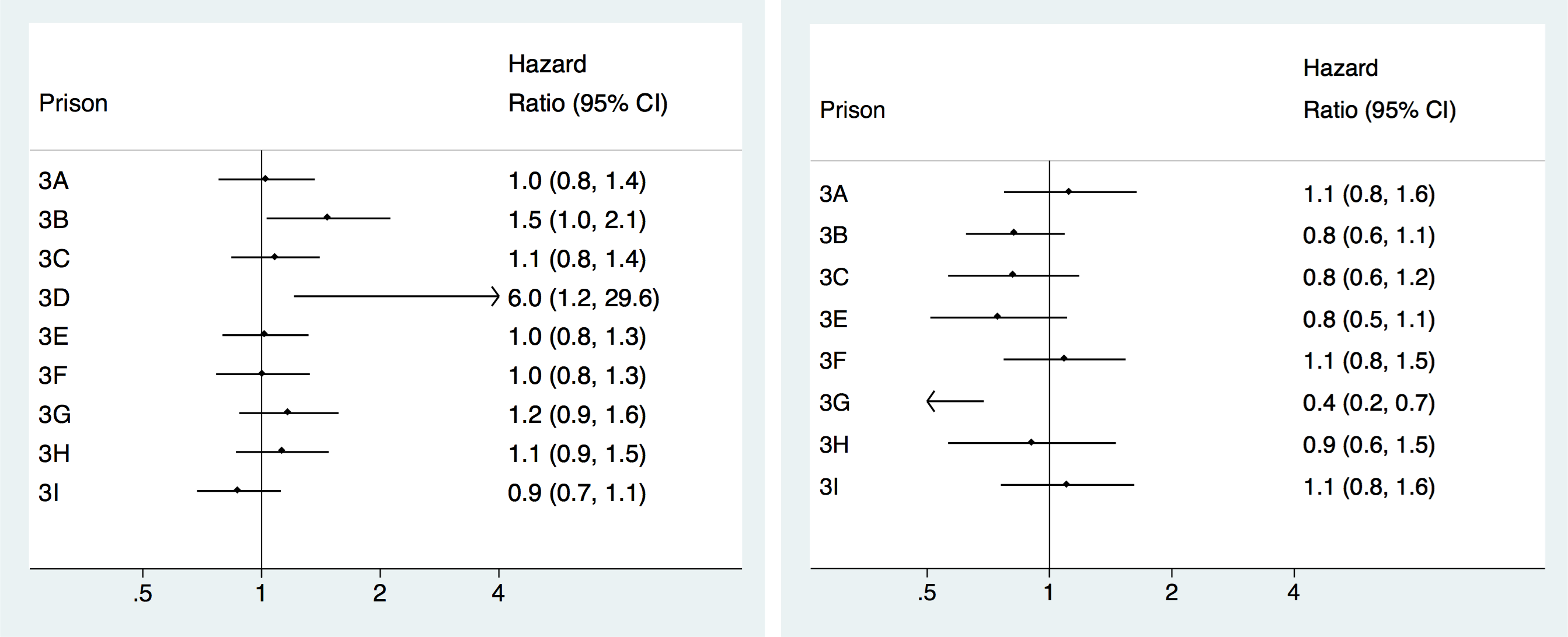

Supplement: S3 Fig — A. Between-individual analyses of recidivism risk among prisoners released from low security prisons (level 3): Non-violent reoffending. B. Within-individual analyses of recidivism risk among prisoners released from low security prisons (level 3): Non-violent reoffending. (TIF) [file pone.0267941.s003.tif]

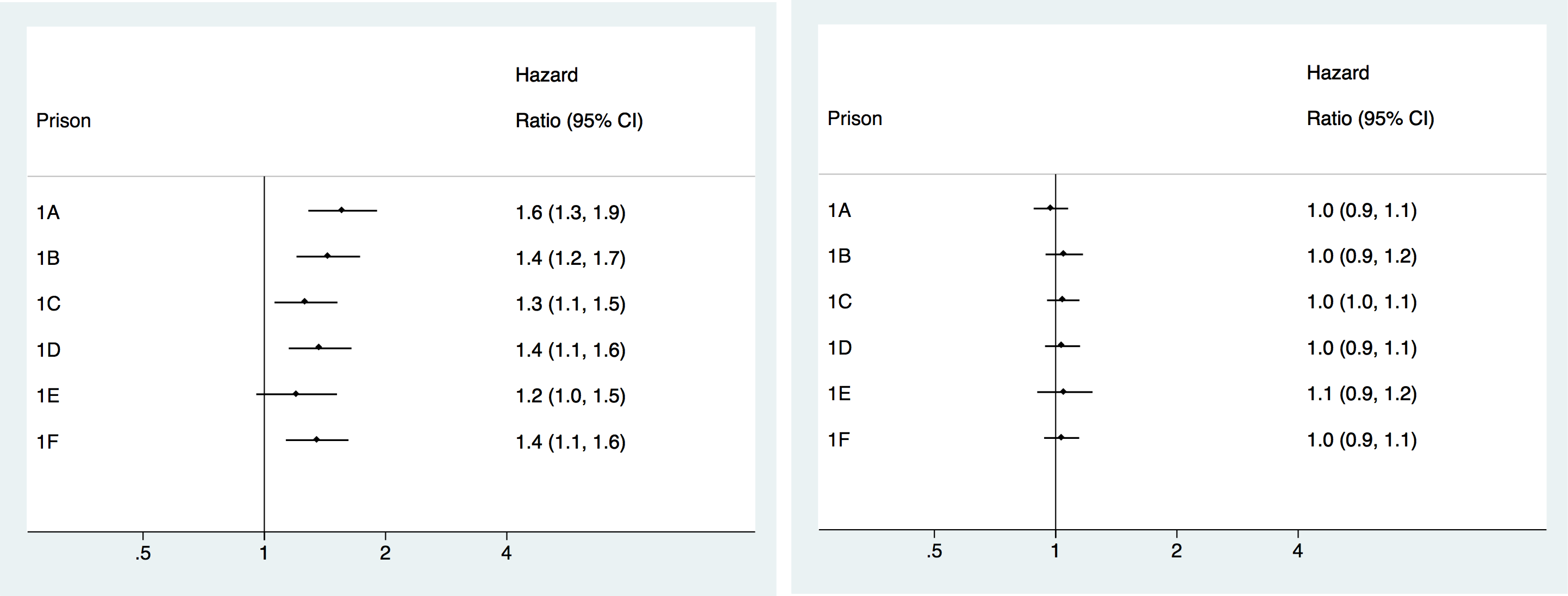

Supplement: S4 Fig — A. Between-individual analyses of recidivism risk among prisoners released from high security prisons (level 1): Violent reoffending. B. Within-individual analyses of recidivism risk among prisoners released from high security prisons (level 1): Violent reoffending. (TIF) [file pone.0267941.s004.tif]

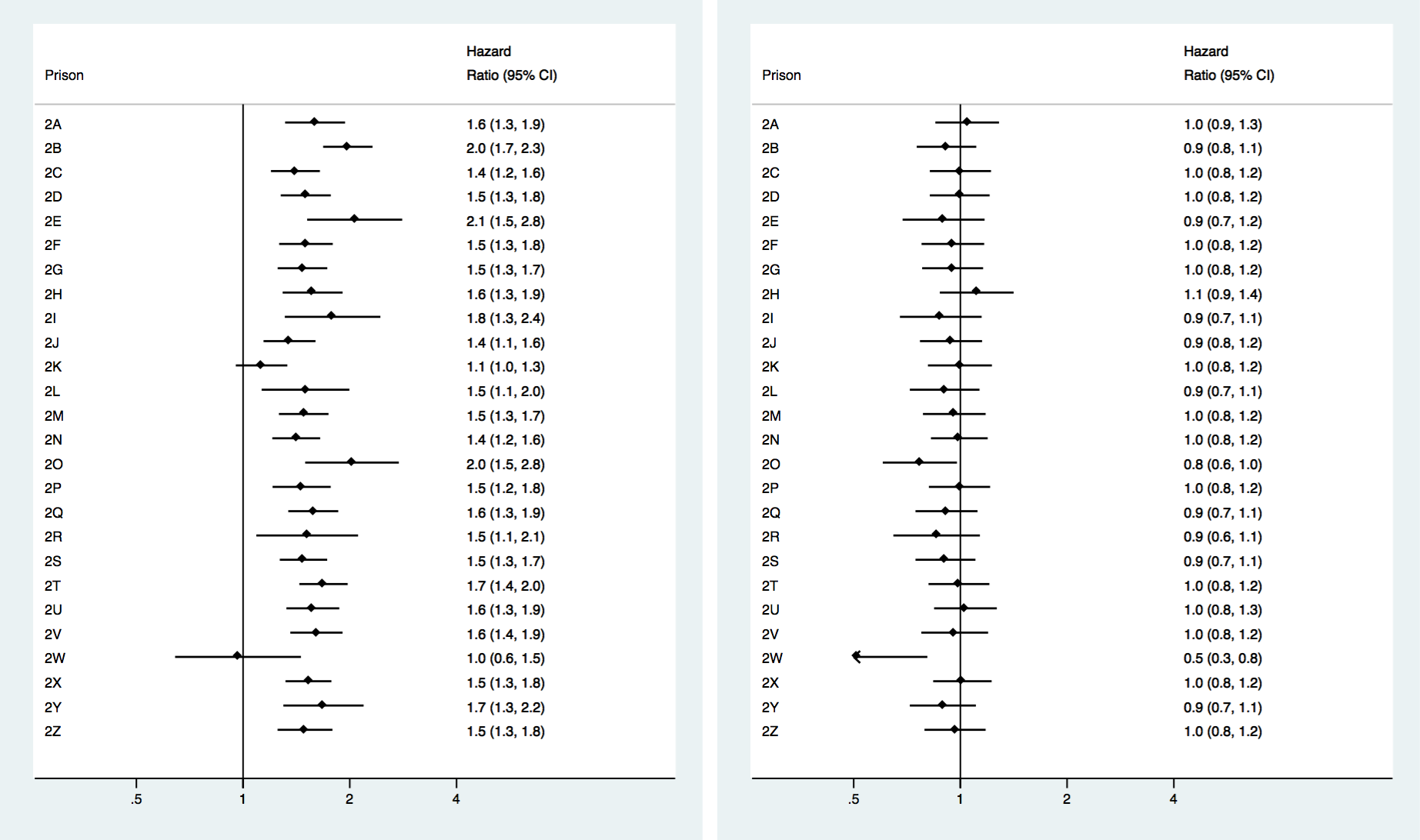

Supplement: S5 Fig — A. Between-individual analyses of recidivism risk among prisoners released from medium security prisons (level 2): Violent reoffending. B. Within-individual analyses of recidivism risk among prisoners released from medium security prisons (level 2): Violent reoffending. (TIF) [file pone.0267941.s005.tif]

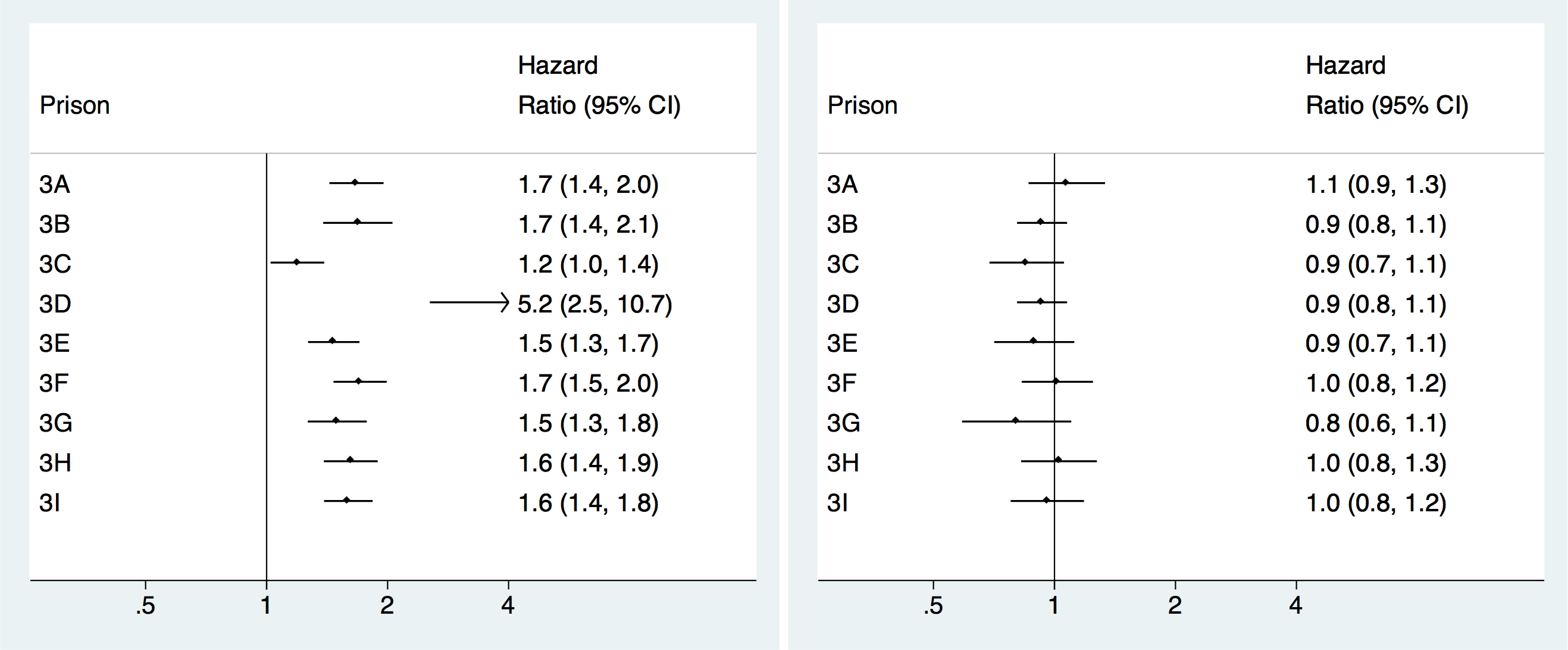

Supplement: S6 Fig — A. Between-individual analyses of recidivism risk among prisoners released from low security prisons (level 3): Violent reoffending. B. Within-individual analyses of recidivism risk among prisoners released from low security prisons (level 3): Violent reoffending. (TIF) [file pone.0267941.s006.tif]

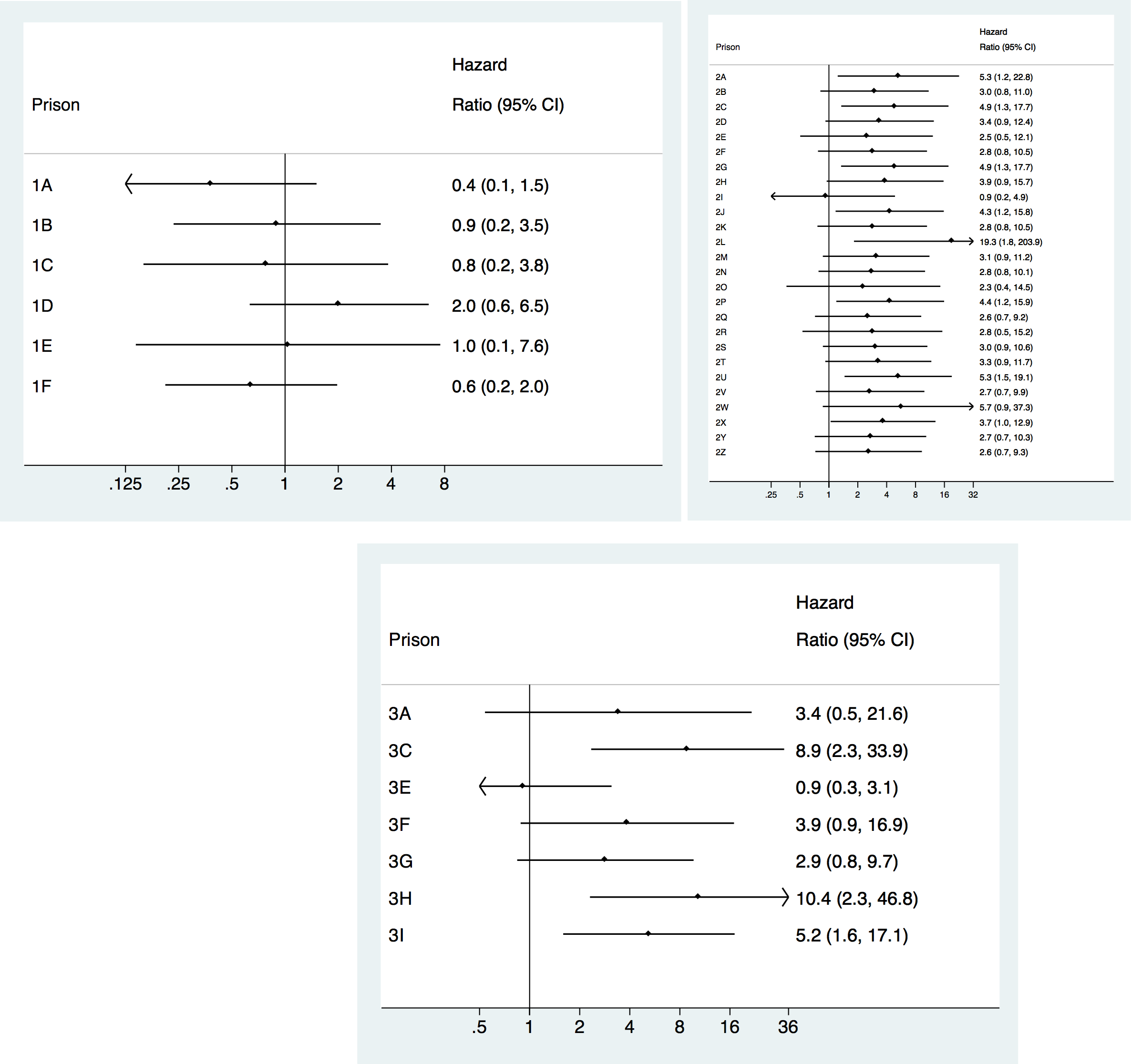

Supplement: S7 Fig — A. Within-individual analyses of recidivism risk among prisoners released from high security prisons (level 1); custodial sentences. B. Within-individual analyses of recidivism risk among prisoners released from medium security prisons (level 2); custodial sentences. C. Within-individual analyses of recidivism risk among prisoners released from low security prisons (level 3); custodial sentences. (TIF) [file pone.0267941.s007.tif]

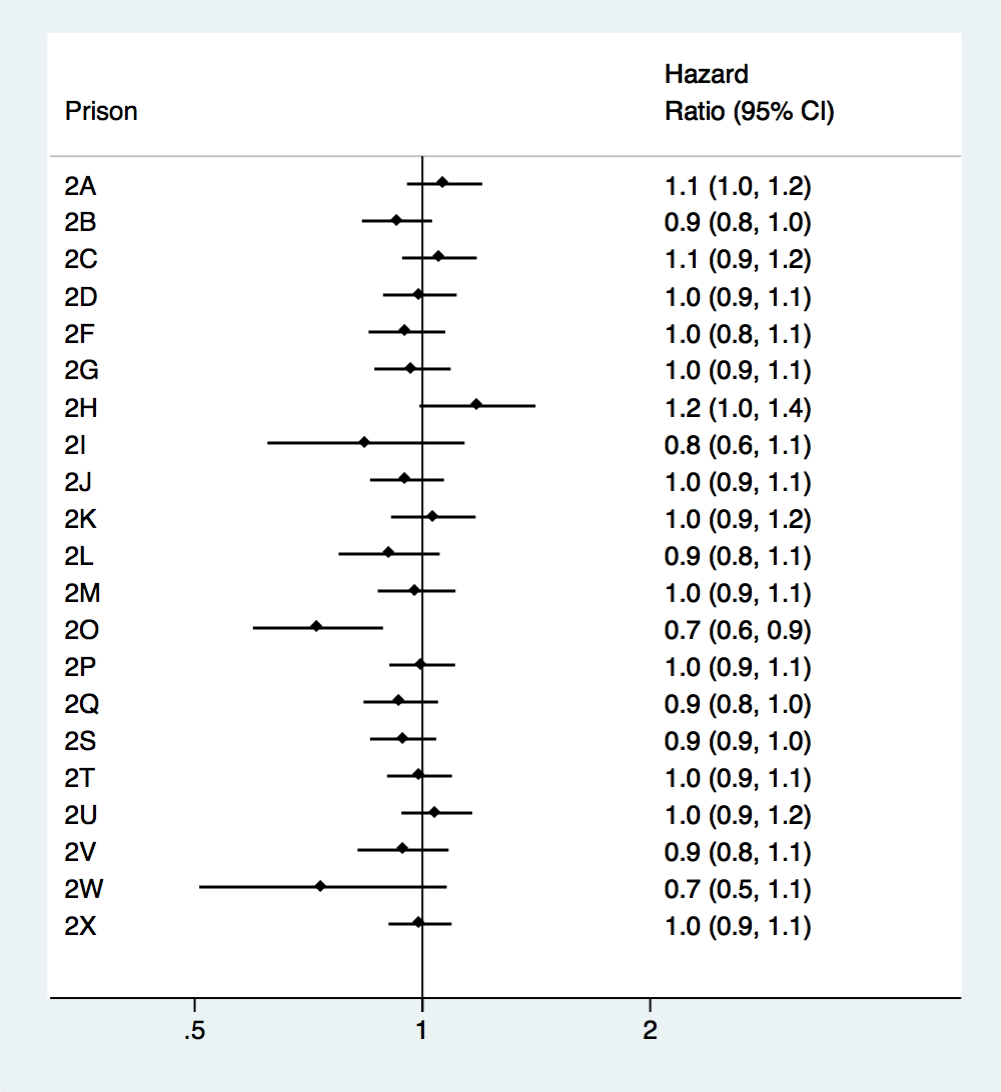

Supplement: S8 Fig — (TIF) [file pone.0267941.s008.tif]

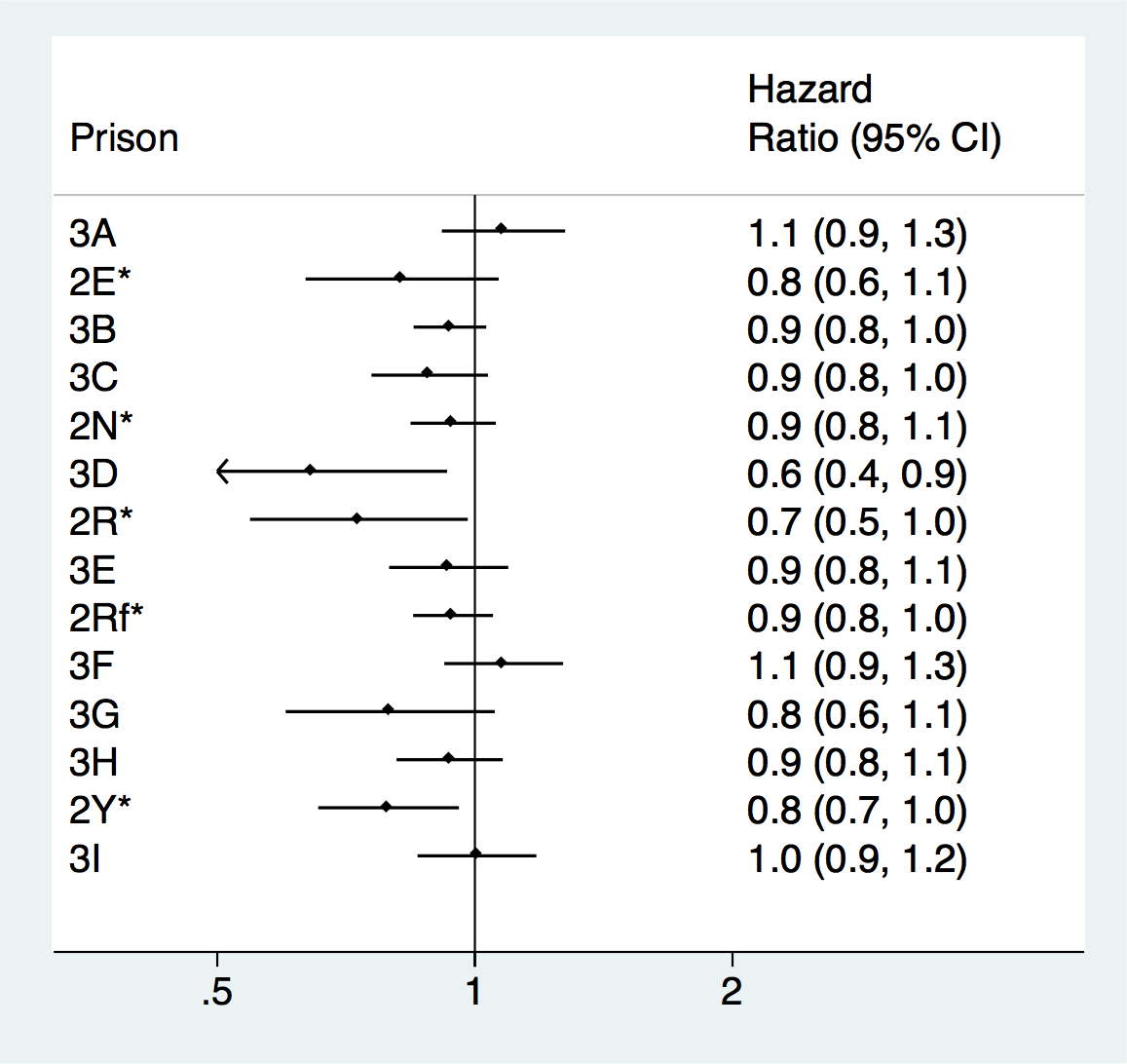

Supplement: S9 Fig — Note: * The prisons that have both security levels 2 and 3. (TIF) [file pone.0267941.s009.tif]

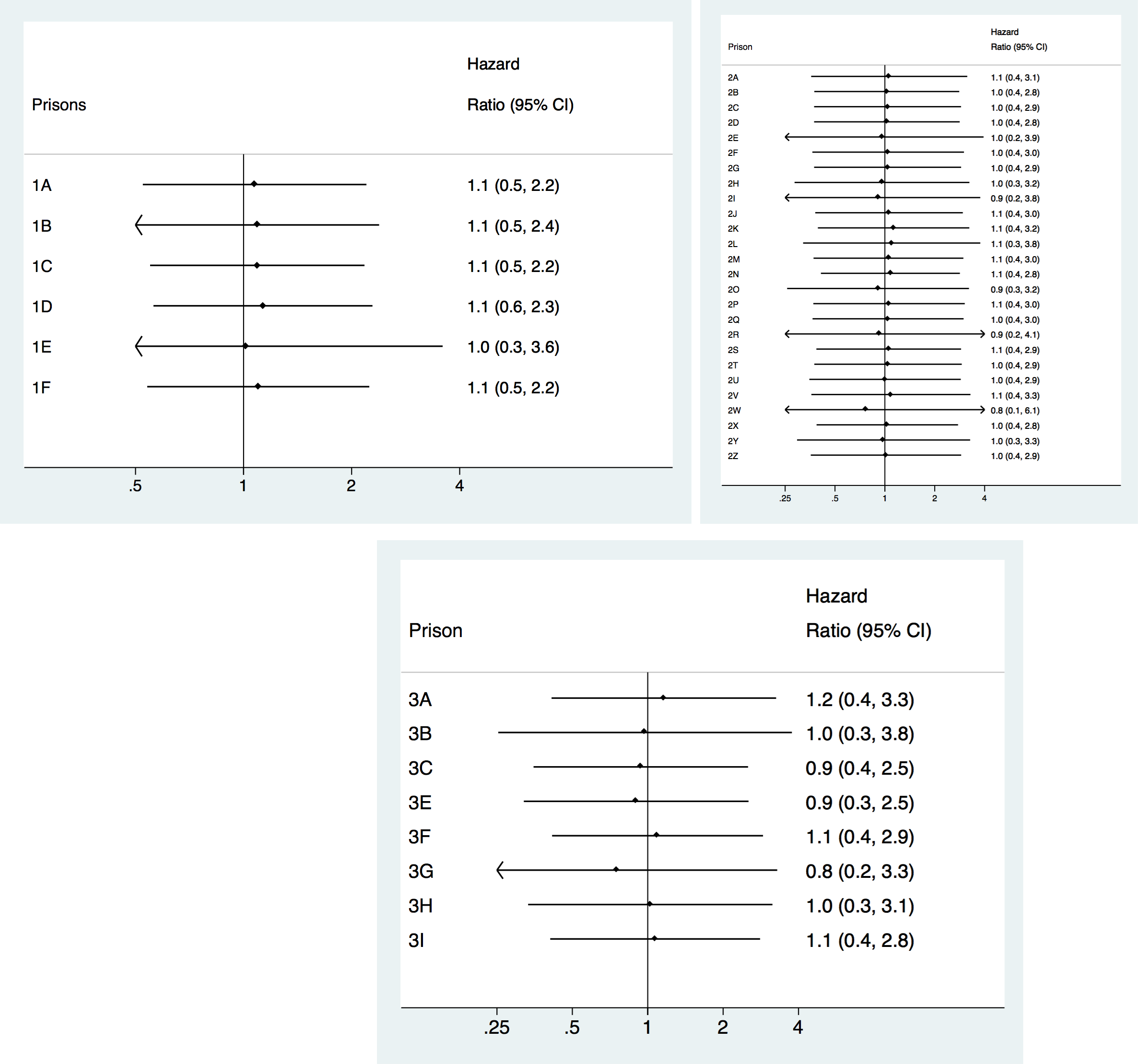

Supplement: S10 Fig — A. Within-individual analyses of recidivism risk among prisoners released from high security prisons (level 1); excluding cloned sentences. B. Within-individual analyses of recidivism risk among prisoners released from medium security prisons (level 2); excluding cloned sentences. C. Within-individual analyses of recidivism risk among prisoners released from low security prisons (level 3); excluding cloned sentences. (TIF) [file pone.0267941.s010.tif]
