# Supplementary material for: Associations between prisons and recidivism: A nationwide longitudinal study
Source: PLoS One. 2022 May 17;17(5):e0267941. doi: 10.1371/journal.pone.0267941 (PMC9113604; doi:10.1371/journal.pone.0267941)
Supplement: S1 Table — (DOCX) [file pone.0267941.s011.docx]

**S1 Table.** Characteristics of individuals with one exposure (i.e. placement in one prison) vs. multiple exposures (i.e. placement in different prisons) within the same security level during 2006 to 2013 in Sweden.

|  | One high security prison exposure (level 1)  (n = 3,752) | Several high security prison exposures (level 1)  (n = 2,098) |
| --- | --- | --- |
| Male | 98.0% (3,677) | 100.0% (2,098) |
| Female | 2.0% (75) | 0.0% (0) |
| Age at the start of prison sentence | 35.4 (11.9) | 31.8 (10.1) |
| Alcohol use disorder | 34.1% (1,279) | 35.9% (753) |
| Drug use disorders | 55.0% (2,064) | 65.4% (1,372) |
| Psychiatric disorders | 13.5% (506) | 15.1% (316) |
|  | | |
|  | One medium security prison exposure (level 2)  (n = 16,375) | Several medium security prison exposures (level 2)  (n = 7,775) |
| Male | 87.7% (14,358) | 91.5% (7,117) |
| Female | 12.3% (2,017) | 8.5% (658) |
| Age at the start of prison sentence | 36.2 (12.0) | 33.6 (11.0) |
| Alcohol use disorder | 32.7% (5,353) | 43.4% (3,376) |
| Drug use disorders | 51.8% (8,484) | 75.7% (5,869) |
| Psychiatric disorders | 12.4% (2,027) | 18.3% (1,421) |
|  | | |
|  | One low security prison exposure (level 3)  (n = 14,469) | Several low security prison exposures (level 3)  (n = 2,187) |
| Male | 96.1% (15,824) | 99.5% (2,176) |
| Female | 3.9% (645) | 0.5% (11) |
| Age at the start of prison sentence | 40.5 (13.7) | 39.2 (12.5) |
| Alcohol use disorder | 32.0% (5,271) | 34.5% (754) |
| Drug use disorders | 25.5% (4,196) | 27.7% (605) |
| Psychiatric disorders | 7.7% (1,271) | 6.8% (149) |
