# Supplementary material for: Associations between prisons and recidivism: A nationwide longitudinal study
Source: PLoS One. 2022 May 17;17(5):e0267941. doi: 10.1371/journal.pone.0267941 (PMC9113604; doi:10.1371/journal.pone.0267941)
Supplement: S2 Table — * Reference category: Male; ** Only time-varying individual-level covariates (i.e. factors that may change during follow-up) are entered in the within-individual analyses. (DOCX) [file pone.0267941.s012.docx]

**S2 Table.** Covariate estimates in between- and within-individual analyses of recidivism risk among prisoners released from high, medium, and low security prisons (levels 1-3).

| Covariates | Between-individual analyses of recidivism risk among prisoners released from high security prisons (level 1) | Within-individual analyses of recidivism risk among prisoners released from high security prisons (level 1) |
| --- | --- | --- |
| Sex* | 0.76 (0.25-2.33) | n/a** |
| Age at the start of prison sentence | 0.97 (0.96-0.97) | 0.79 (0.75-0.84) |
| Number of previous convictions | 2.16 (1.93-2.43) | 0.83 (0.55-1.26) |
| Imprisonment length | 1.00 (0.99-1.00) | 0.99 (0.99-0.99) |
| Alcohol use disorder | 1.05 (0.95-1.17) | 1.13 (0.88-1.44) |
| Drug use disorders | 1.47 (1.39-1.56) | 0.94 (0.79-1.13) |
| Psychiatric disorders | 1.09 (0.94-1.26) | 1.00 (0.84-1.44) |
|  | Between-individual analyses of recidivism risk among prisoners released from medium security prisons (level 2) | Within-individual analyses of recidivism risk among prisoners released from medium security prisons (level 2) |
| Sex* | 0.65 (0.50-0.84) | n/a** |
| Age at the start of prison sentence | 0.99 (0.98-0.99) | 0.82 (0.80-0.83) |
| Number of previous convictions | 1.72 (1.64-1.80) | 0.91 (0.81-1.02) |
| Imprisonment length | 0.99 (0.99-0.99) | 0.99 (0.99-0.99) |
| Alcohol use disorder | 1.10 (1.06-1.15) | 0.91 (0.84-0.99) |
| Drug use disorders | 1.53 (1.49-1.57) | 1.01 (0.96-1.07) |
| Psychiatric disorders | 1.02 (0.96-1.08) | 0.99 (0.90-1.10) |
|  | Between-individual analyses of recidivism risk among prisoners released from low security prisons (level 3) | Within-individual analyses of recidivism risk among prisoners released from low security prisons (level 3) |
| Sex* | 0.31 (0.16-0.62) | n/a** |
| Age at the start of prison sentence | 0.98 (0.98-0.98) | 0.76 (0.73-0.80) |
| Number of previous convictions | 1.76 (1.65-1.88) | 0.88 (0.68-1.15) |
| Imprisonment length | 0.99 (0.99-1.00) | 0.99 (0.99-1.00) |
| Alcohol use disorder | 1.18 (1.11-1.26) | 1.02 (0.84-1.25) |
| Drug use disorders | 1.51 (1.44-1.58) | 0.97 (0.83-1.14) |
| Psychiatric disorders | 1.09 (0.97-1.23) | 0.95 (0.66-1.37) |

* Reference category: male; ** Only time-varying individual-level covariates (i.e. factors that may change during follow-up) are entered in the within-individual analyses
